# 3D-printed long-acting 5-fluorouracil implant to prevent conjunctival fibrosis in glaucoma

**DOI:** 10.1093/jpp/rgac100

**Published:** 2023-01-06

**Authors:** Nicole Ioannou, Jinyuan Luo, Mengqi Qin, Matteo Di Luca, Essyrose Mathew, Aristides D Tagalakis, Dimitrios A Lamprou, Cynthia Yu-Wai-Man

**Affiliations:** Faculty of Life Sciences & Medicine, King’s College London, London, UK; Faculty of Life Sciences & Medicine, King’s College London, London, UK; Department of Ophthalmology, Renmin Hospital of Wuhan University, Wuhan, China; Faculty of Life Sciences & Medicine, King’s College London, London, UK; School of Pharmacy, Queen’s University Belfast, Belfast, UK; School of Pharmacy, Queen’s University Belfast, Belfast, UK; Department of Biology, Edge Hill University, Ormskirk, UK; School of Pharmacy, Queen’s University Belfast, Belfast, UK; Faculty of Life Sciences & Medicine, King’s College London, London, UK

**Keywords:** 3D printing, 5-FU, glaucoma, implant, drug delivery, fibrosis

## Abstract

**Objectives:**

To develop a sustained release 5-fluorouracil (5-FU) implant by three-dimensional (3D) printing to effectively prevent conjunctival fibrosis after glaucoma surgery.

**Methods:**

3D-printed implants composed of polycaprolactone (PCL) and chitosan (CS) were fabricated by heat extrusion technology and loaded with 1% 5-FU. Light microscopy and scanning electron microscopy were used to study the surface morphology. The 5-FU concentration released over 8 weeks was measured by ultraviolet visible spectroscopy. The effects on cell viability, fibroblast contractility and the expression of key fibrotic genes were assessed in human conjunctival fibroblasts.

**Key findings:**

The PCL–CS-5-FU implant sustainably released 5-FU over 8 weeks and the peak concentration was over 6.1 μg/ml during weeks 1 and 2. The implant had a smooth surface and its total weight decreased by 3.5% after 8 weeks. The PCL–CS–5-FU implant did not affect cell viability in conjunctival fibroblasts and sustainably suppressed fibroblast contractility and key fibrotic genes for 8 weeks.

**Conclusions:**

The PCL–CS–5-FU implant was biocompatible and degradable with a significant effect in suppressing fibroblast contractility. The PCL–CS–5-FU implant could be used as a sustained release drug implant, replacing the need for repeated 5-FU injections in clinic, to prevent conjunctival fibrosis after glaucoma surgery.

## Graphical Abstract

**Graphical Abstract:**
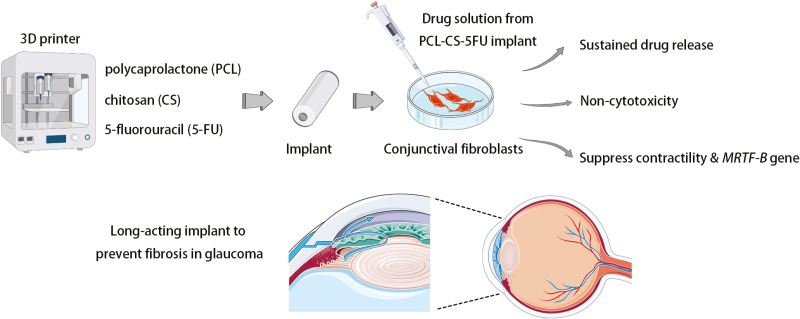

## Introduction

Glaucoma, a group of optic neuropathies that present with progressive excavation of the optic disc, is the leading cause of irreversible blindness worldwide. Glaucoma affects more than 75 million patients in the world, and its global prevalence is estimated to increase to 111.8 million cases by 2040.^[1]^ Despite the fact that the precise underlying pathogenesis of glaucoma is not fully understood, abnormal elevation of intraocular pressure (IOP) has been reported to be a primary risk factor associated with the progression of visual field deterioration in glaucoma.^[2, 3]^ Trabeculectomy is a penetrating filtration procedure that creates a permanent fistula connecting the anterior chamber and subconjunctival space for aqueous humour outflow.^[4]^ Although this surgical treatment has been regarded as the gold standard surgical glaucoma treatment to lower IOP, a five-year follow-up study showed that patients who underwent trabeculectomy have a high failure rate of 46.9%.^[5]^ Postoperative fibrosis is the most common cause of trabeculectomy failure,^[6, 7]^ and antifibrotic agents, such as 5-fluorouracil (5-FU) and mitomycin C (MMC), are used to prevent postoperative scarring.^[8, 9]^

5-FU is an antimetabolite used to suppress the wound healing response and is repeatedly injected subconjunctivally after trabeculectomy to suppress subconjunctival scarring and hence to increase the success rate of glaucoma filtering surgery.^[9]^ However, the repeated subconjunctival injections can result in discomfort and a risk of infection in patients, and large cumulative doses of antimetabolites can also lead to adverse side effects, such as hypotony and severe infection.^[10, 11]^ There is therefore an unmet clinical need to design a sustained drug delivery system (DDS) to progressively release antifibrotic agents at therapeutic levels and to prevent conjunctival fibrosis in the long-term perspective.

In light of the minimally invasive surgery developments in ophthalmology, the implantable DDSs, such as Allergan’s Durysta (Allergan plc, Dublin, Ireland) and iDose (Glaukos), have progressed significantly the management of glaucoma patients.^[12]^ Compared to the conventional ocular drug delivery, such as subconjunctival injections and topical drug administration, the drug-eluting implant treatment modalities can increase drug bioavailability and minimise side effects by directly delivering drugs to key sites of action in a sustained and controlled manner.^[13]^

Amongst the several biomedical applications of implanted DDS, three-dimensional (3D) printing (3DP) implants are set to have more promising applications in drug delivery.^[14, 15]^ 3DP is an additive manufacturing technology where the customised objects are manufactured layer-by-layer (LbL) with high precision.^[16, 17]^ As 3D-printed medical implants can personalise the medical device geometries as well as the drug release rate, it will have promising results when applied to ophthalmology.^[15]^

To the best of our knowledge, only a few studies have been carried out to develop a 5-FU sustained release implant. This study used 3D-printed technology to develop a long-acting implant loaded with 1% 5-FU in a polymer mixture of polycaprolactone (PCL) and chitosan (CS). PCL is a cost-effective and Food and Drug Administration-approved degradable polyester for a variety of applications, which has been extensively studied in tissue engineering and for drug delivery.^[18]^ PCL has high mechanical strength and biaxial stretching property, which enable it to be extremely thin (<10 μm in thickness) and as a result, the implant can be more flexible and comfortable.^[19, 20]^ On the other hand, CS is a bioactive polymer that is widely used in medicine due to its functional properties, such as biodegradability, biocompatibility, antimicrobial properties and minimal side effects.^[21–23]^ It has been shown that PCL composite sponges mixed with CS could significantly enhance the 5-FU effect of inhibiting the proliferation of human head and neck squamous cell carcinoma cell lines without influencing its role in inhibiting cancer cell migration.^[24]^

In this study, we developed a 5-FU-sustained release implant with good biocompatibility. The sustained release of 5-FU enables this DDS to become a potentially useful approach to reduce surgical failure due to postoperative fibrosis and to improve the long-term surgical outcomes in glaucoma patients.

## Materials and Methods

### Materials

Polycaprolactone powder (MW 50000 Daltons; Tm = 58°C; PCL) was supplied by Polysciences Inc. The low molecular weight chitosan powder (deacetylation degree ≥ 75%; Tm = 102.5°C; CS) and the 5-Fluorouracil (MW 130.08 g/mol; sparingly soluble in water <1 mg/ml; ≥ 99% HPLC; Tm = 282°C; 5-FU) were purchased from Sigma-Aldrich.

### Manufacturing of 3D-printed implants

Three 3D-printed implants (PCL–CS–5-FU, PCL–CS and PCL) were designed by Tinkercad and manufactured using the BIOX Bioprinter (CELLINK, Sweden) equipped with Thermoplastic Printhead.^[25]^ The system integrates a Clean Chamber Technology, a dual high-powered fans channel air, and UV-C germicidal lights, allowing the removal of nearly 100% of unwanted particles and microorganisms to achieve a sterile implantable device. The implant containing only PCL was fabricated following the Cellink Printing Protocol. To ensure the homogeneity of the polymers and drug mixture, the polymers were vortexed five times for 60 s each time prior to printing. The polymer powders were then directly transferred into the stainless-steel cartridge without adding any solvent and a 22G nozzle (0.413 mm internal diameter) was used for the PCL implant. The heat extrusion was set at 180°C and the pressure was 175 kPa. The cannula length was 14.5 cm and the printing speed was 2 mm/s.

The PCL–CS and PCL–CS–5-FU implants were manufactured using a mixture of PCL and CS. A preliminary screening was carried out to determine the best polymer combination in terms of flow and printability, and the ratio of 30:1 for PCL:CS w/w was chosen. The implants were printed with dimensions of *l* = 21.0 mm and *h* = 1.2 mm. The 5-FU drug was formulated as 1% of the total weight of the implant. The heat extrusion was set at 130°C and the pressure was 195 kPa, and a 18G nozzle (0.838 mm internal diameter) was used with the printing speed set at 3.5 mm/s.

### Light microscopy imaging

Light microscopy images of the implants were taken using an Olympus CKX41 inverted microscope with Olympus CellSens Standard 1.13 (Build 13479) software at 4×, 10× and 20× magnification.

### Scanning electron microscopy (SEM) imaging

The surface morphology of the 3D printing filaments was examined using a Quanta 200 Emission Electron Microscope (FEI Company, USA) with a 10 nm gold coating layer. SEM micrographs were taken at an operating voltage of 5.0 kV at a magnification of 2000× and 8000×.

### *In vitro* drug release studies

About 20 mg of each implant was weighed and placed in a 3 cm piece of SpectraPor Biotech Grade Dialysis Membrane (Repligen, USA). The dialysis membranes were folded in half and a clip was placed to secure both ends. Each dialysis membrane was then placed in a 25 ml flask containing 25 ml of milliQ water and a magnet.^[26, 27]^ Parafilm was used to cover the top of the flasks. The three flasks were placed on a magnetic stirrer and left for 8 weeks with continued stirring at room temperature (RT). At the end of each week, 5 ml of samples were collected from each flask and stored in a -20°C freezer. 5 ml of milliQ water was then added to the flasks for replacement.

Ultraviolet visible (UV) spectroscopy was performed on each sample to measure the concentration of 5-FU and the drug release rate over 8 weeks. The UV spectrum was recorded in triplicates using a 7205 Jenway UV–VIS scanning spectrophotometer (Cole-Parmer, Staffordshire, UK), and the absorbance was at a wavelength of 266 nm.

### Primary cell culture

Human conjunctival fibroblasts were cultured from conjunctival samples collected from glaucoma patients after informed consent. The fibroblasts were maintained in complete media [DMEM (Gibco, Thermo Scientific, UK), 10% foetal calf serum (FCS), 100 U/ml penicillin, 100 mg/ml streptomycin] and incubated at 37°C with 5% CO_2_ and 95% humidity. Fibroblasts between passages 6 and 9 were used in the experiments. All experiments were carried out according to the rules of the Declaration of Helsinki and approved by the West of Scotland Research Ethics Committee (REC 19/WS/0146, date of approval 2/10/2019).

### Cell viability assay

Human conjunctival fibroblasts were plated in a 96-well plate at a density of 0.625 × 10^4^ cells per well. The cells were treated with media containing 300 μl of the drug solution collected from each implant in week 1 to week 8 or with no drug control. For the 5-FU treatment, 5-FU was added for 5 minutes, the cells were washed with PBS, followed by the addition of 400 μl of complete media. After 24-h treatment, the medium was removed and replaced with 100 μl of fresh complete media. For each condition, independent triplicates were performed.

A colorimetric assay was used to measure cell viability. 20 μl of CellTiter 96 Aqueous One solution (Promega, Southampton, UK) were added to each well. The plate was incubated for 2 h and the absorbance was measured at 490 nm using the PHERAstar FS instrument (BMG Labtech, Aylesbury, UK). Cell viability was calculated as a percentage of the viability of the no drug control cells.

### Collagen contraction assay

A cell suspension of 1 × 10^5^ conjunctival fibroblasts was centrifuged at 1500 rpm for 5 min and the cell pellet was resuspended in 100 μl of FCS. The collagen gel mix solution was prepared as previously described.^[28]^ To prepare the collagen gel mix solution, 1 ml Type I collagen (First Link UK Ltd, Wolverhampton, UK) was combined with 160 μl of concentrated medium consisting of DMEM X10 (Sigma-Aldrich, Gillingham, UK), sodium bicarbonate 7.5% solution and 2 mM l-Glutamine (Thermo Scientific, Loughborough, UK). Sodium hydroxide 1 M was added to adjust the pH to 7.0. The cells were then mixed with the collagen solution and 150 μL of the collagen mix solution were added in each Mattek dish. The gels were placed in the incubator for 10 min to set.

About 3.375 ml of each implant solution from the first 4 weeks was added to 1.125 ml complete media to test their effects on fibroblast contraction. Once the gels were polymerised, 1.5 ml of the drug media or no drug control were added. For the 5-FU treatment, 5-FU was added for 5 min, the cells were washed, and 1.5 mL of complete media were then added. The gels were gently detached from the well and put back into the incubator. Gel photos were taken daily over 7 days and analysed using the ImageJ program. The percentage of matrix contraction was calculated using the formula: [(Area of gel at Day 0—Area of gel at Day *n*)/Area of gel at Day 0] × 100.

### Real-time quantitative PCR

Human conjunctival fibroblasts were plated in 6-well plates at a density of 1 × 10^5^ cells per well. The cells were treated for 24 h with 1.5 ml of media containing the drug solution collected from each implant from week 1 to week 8 or with no drug control. For the 5-FU treatment, 5-FU was added for 5 min, the cells were washed, and 1.5 ml of complete media were then added. Each condition was performed as independent triplicates.

RNA was extracted using the QIAGEN Quick-Start RNeasy Mini Kit (QIAGEN GmbH, Hilden, Germany) protocol and cDNA was synthesised according to the cDNA reverse transcription kit (Applied Biosystems, Loughborough, UK). A ViiA7 Real-Time PCR system (ThermoFisher Scientific, Loughborough, UK) was used for the RT-qPCR assay. The primers used for RT-qPCR are shown in Table 1. The plate was run for 40 cycles and each cycle setup consisted of: Holding stage: 50°C for 2 min and 95°C for 5 min; PCR stage: 95°C for 5 min and 60°C for 30 s. The 2^-ΔΔCT^ method was used to analyse the data.

**Table 1:** Primers used for RT-qPCR

Gene	Forward primer	Reverse primer
_*ACTA2*_	AATGCAGAAGGAGATCACGC	TCCTGTTTGCTGATCCACATC
_*COL1A2*_	TGGATGAGGAGACTGGCAAC	TTAGAACCCCCTCCATCCCAC
_*CTGF*_	CAGAGTGGAGCGCCTGTT	CTGCAGGAGGCGTTGTCA
_*GAPDH*_	ACGGATTTGGTCGTATTGGGC	TTGACGGTGCCATGGAATTTG
_*MRTF-B*_	CTTCCTGTGGACTCCAGTG	TGTGACTCCTGACTCGCAG

### Statistical analysis

All data are expressed as mean ± SEM. Statistical analysis was carried out using One-way ANOVA followed by post hoc test. Statistically significant values were represented as: ^*^, *p* < 0.05; ^**^, *p* < 0.01; ^***^, *p* < 0.001; ^****^, *p* < 0.0001.

## Results

### Light microscopy and SEM analyses of 3D-printed implants

**Figure 1:**
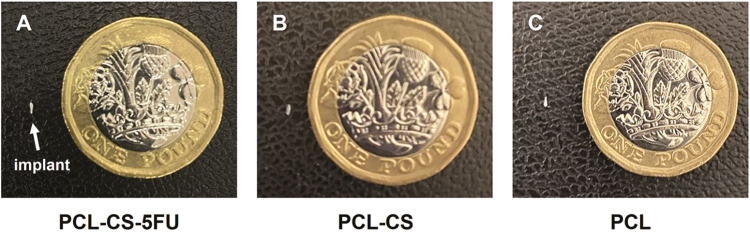
Photographs of 3D-printed implants (white arrow) versus a pound sterling coin for size comparison. (A) PCL–CS–5-FU; (B) PCL–CS and (C) PCL.

**Figure 2:**
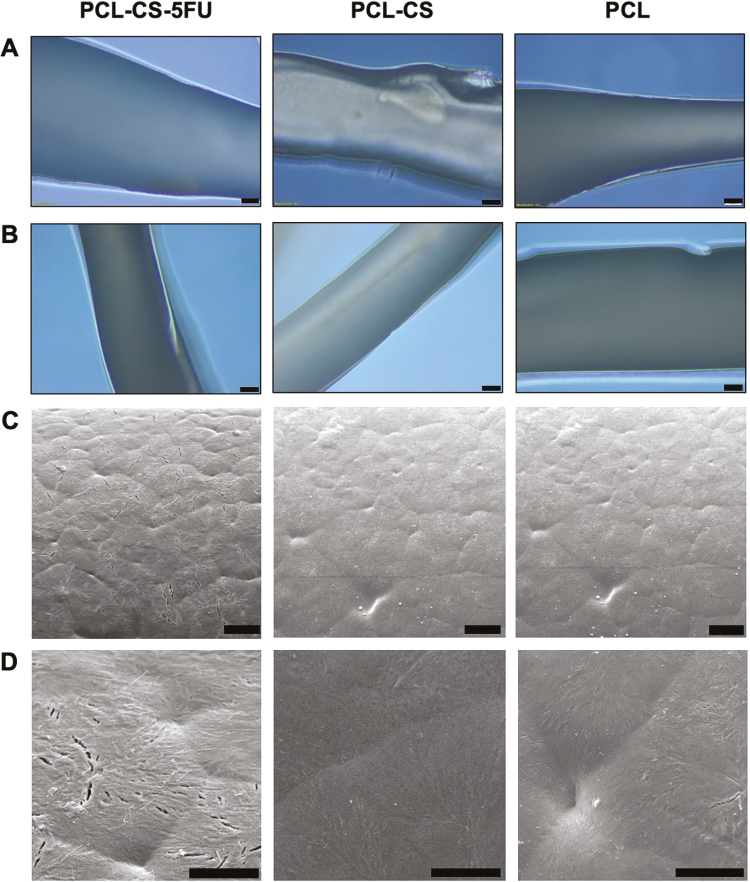
Light microscopy images and SEM micrographs of PCL–CS–5-FU (left), PCL–CS (middle) and PCL (right) implants. Light microscopy images (A) before drug release experiment and (B) after drug release experiment; Scale bar, 50 μm. SEM micrographs were taken at magnifications of (C) 2000× and (D) 8000×; Scale bar, 10 μm.

Figure 1 shows the size comparison between the three different 3D-printed implants versus a pound sterling coin. The implants were white, and light microscopy showed smooth surfaces of the PCL–CS–5-FU, PCL–CS, and PCL implants before the drug release experiment (Figure 2A). There were no significant morphological differences between the implants after 8 weeks of drug release experiment (Figure 2B). The SEM images also showed a smooth surface with no anomalies in the PCL–CS–5-FU, PCL–CS and PCL implants at both 2000× (Figure 2C) and 8000× (Figure 2D) magnifications.

### Sustained release and weight changes of 3D-printed implants

**Figure 3:**
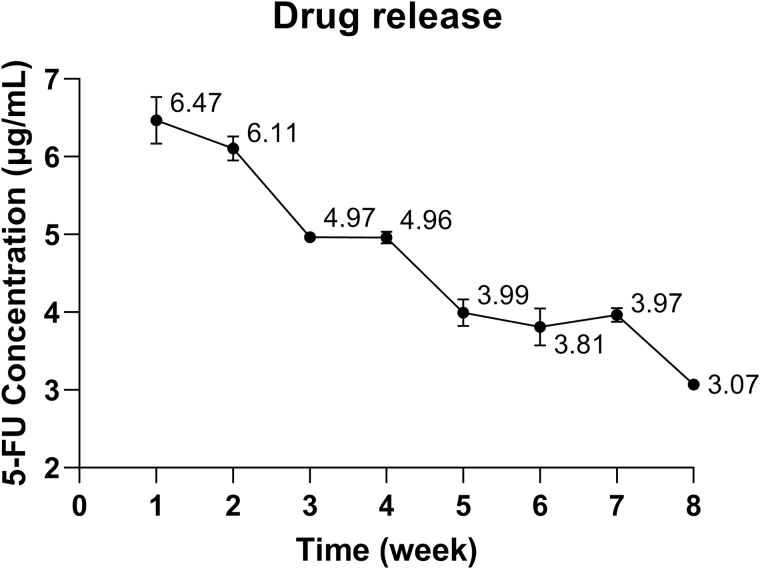
The 5-FU concentration released from the PCL–CS–5-FU implant was measured over 8 weeks using ultraviolet visible spectroscopy. Results represent mean ± SEM, *N* = 3.

UV spectroscopy was used to measure the 5-FU concentration released from the implant over 8 weeks. The drug release concentration peaked at 6.47 μg/ml during week 1 and 6.11 μg/ml during week 2, then decreased to 4.97 and 4.96 μg/ml during weeks 3 and 4, respectively. The 5-FU concentrations during weeks 5, 6 and 7 were 3.99, 3.81 and 3.97 μg/ml, respectively. At week 8, the 5-FU concentration maintained at 3.07 μg/ml (Figure 3).

**Figure 4:**
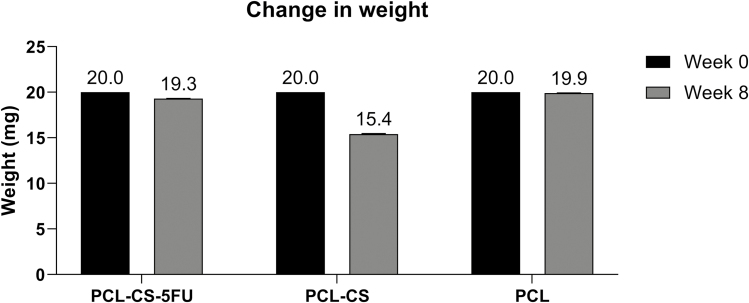
Change in weight of the PCL–CS–5-FU, PCL–CS and PCL implants before and after the drug release experiment. 20.0 mg was used before drug release experiment and results represent mean ± SEM, *N* = 3, after drug release experiment.

All three implants had an initial weight of 20.0 mg. After 8 weeks of drug release experiment, the PCL–CS–5-FU and PCL–CS implants exhibited a weight decrease of 3.5% and 23.0%, respectively (Figure 4). On the other hand, the PCL implant only had a small decrease in weight of 0.5% (Figure 4).

### Effect of 3D-printed implants on cell viability of human conjunctival fibroblasts

**Figure 5:**
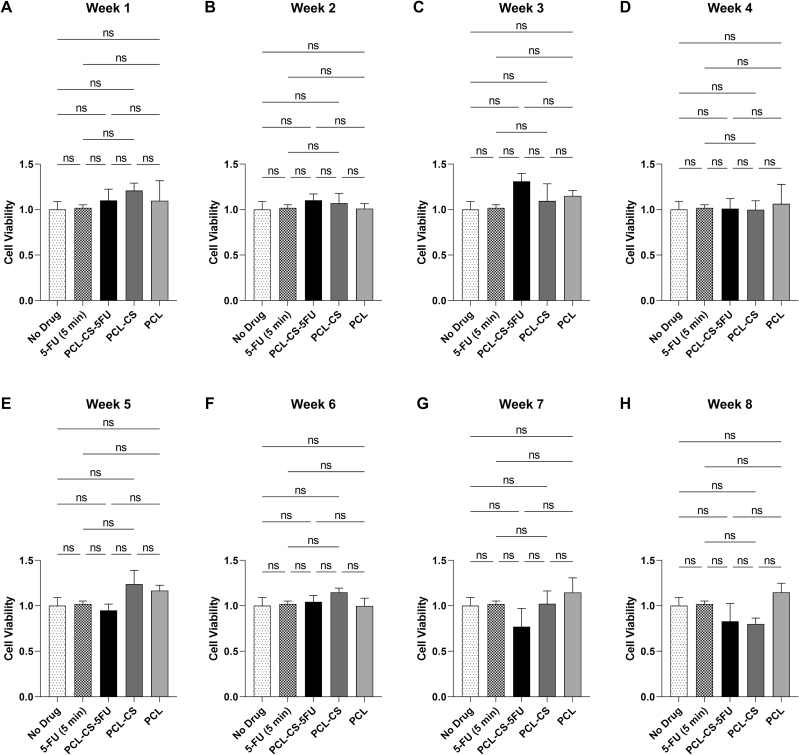
Cell viability of human conjunctival fibroblasts after 1-day treatment with no drug control, 5-FU (5 min) and the drug solutions of the 3D-printed implants collected from (A) week 1, (B) week 2, (C) week 3, (D) week 4, (E) week 5, (F) week 6, (G) week 7 and (H) week 8. The cell viability was normalised against no drug control. Results represent mean ± SEM, *N* = 3. ns, not significant.

We next investigated the effects on the cell viability of human conjunctival fibroblasts of the drug solutions from the three implants, compared to no drug control and 5 min of 5-FU treatment. The drug solutions collected from the three different implants each week over the 8 weeks had no significant effect on cell viability compared to the no drug control and the 5-FU treated cells (Figure 5). There were also no statistically significant differences in cell viability between the three implants each week from weeks 1 to 8 (Figure 5).

### Effect of 3D-printed implants on cell contractility of human conjunctival fibroblasts

**Figure 6:**
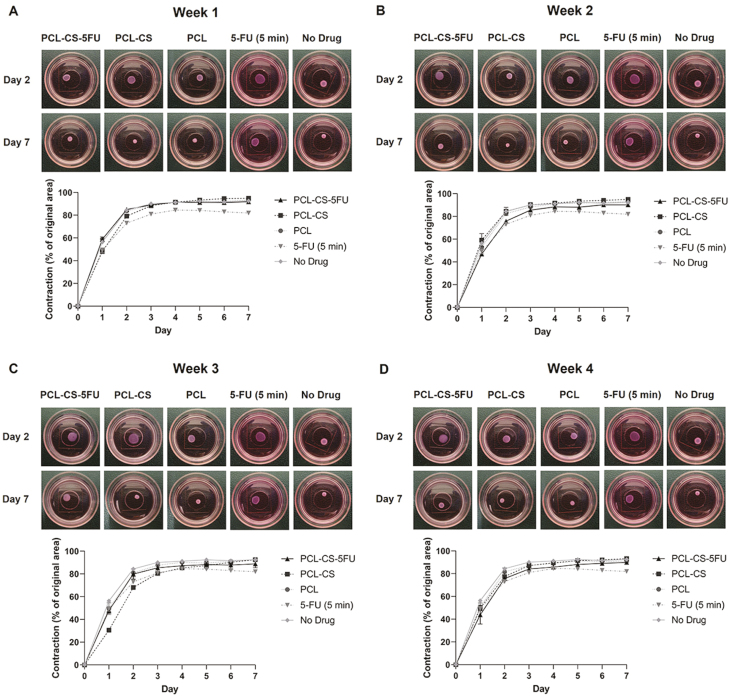
Seven-day collagen contraction assay of human conjunctival fibroblasts after treatment with no drug control, 5-FU (5 min) and the drug solutions of the 3D-printed implants collected from (A) week 1, (B) week 2, (C) week 3 and (D) week 4. Representative gel images on day 2 and day 7 are shown. Results represent mean ± SEM, *N* = 3.

**Figure 7:**
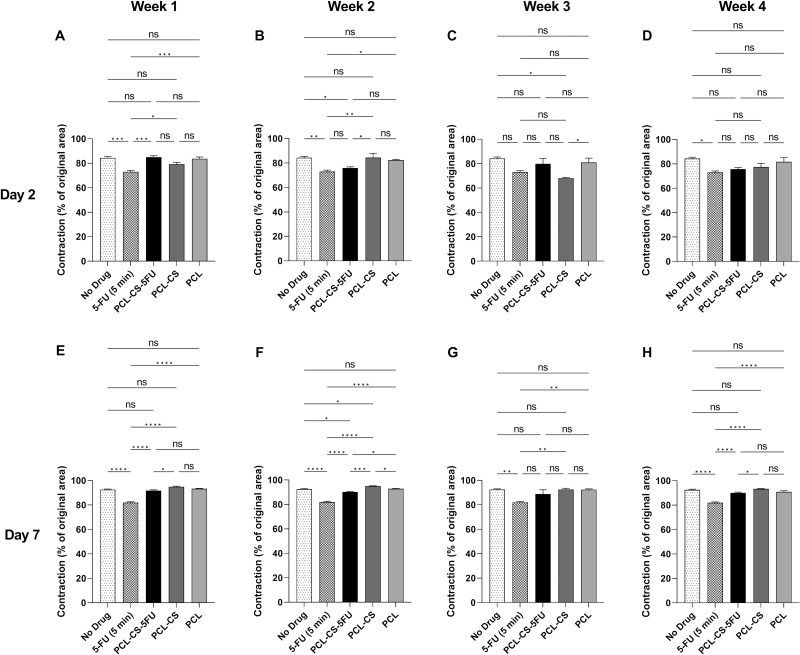
Collagen contraction assay of human conjunctival fibroblasts after treatment with no drug control, 5-FU (5 min) and the drug solutions of the 3D-printed implants collected from week 1 to week 4. The percentage of collagen contraction on day 2 and day 7 was calculated from the original area on day 0. Results represent mean ± SEM, *N* = 3. ns, not significant; ^*^p < 0.05; ^**^p < 0.01; ^***^p < 0.001; ^****^p < 0.0001.

The effects of different drug solutions from week 1 on cell contractility are shown in Figure 6A. All the fibroblast-populated collagen gels had contracted after 7 days, and the percentage contraction increased over the first 2 days and stabilised in the last 5 days. The 5-FU-treated gels had the lowest matrix contraction throughout the 7 days except on day 1. On day 2, the 5-FU treated gels contracted 73.1 ± 1.1%, which was much lower than the no drug control, PCL–CS–5-FU, PCL–CS and PCL-treated gels with the values of 84.4 ± 1.1% (*p* < 0.001), 85.0 ± 1.1% (*p* < 0.001), 79.2 ± 1.6% (*p* = 0.04) and 83.7 ± 1.4% (*p* < 0.001), respectively (Figure 7A). The PCL–CS gel also contracted less than the no drug control, but with no statistical significance (Figure 7A). There was also no statistical significance among the three different implants. On day 7, although no statistical significance was observed between the implants and the no drug control, the PCL–CS–5-FU-treated gels had lower matrix contraction than that of the PCL–CS-treated gels (*p* = 0.01) (Figure 7E).

The effects of different drug solutions from week 2 on cell contractility are presented in Figure 6B. The 5-FU and PCL–CS–5-FU implant showed a decrease in cell contractility compared to the no drug control and the other two implants. On day 2, the PCL–CS–5-FU-treated gels contracted 75.9 ± 0.9%, which was 8.5% (*p* = 0.04), 8.6% (*p* = 0.01) and 6.6% (*p* = 0.14) less than the no drug treatment, PCL–CS and PCL, respectively, but was 2.9% (*p* = 0.79) more than 5-FU treated gels (Figure 7B). A similar trend was observed on day 7, and the PCL–CS–5-FU showed a lower matrix contraction of 90.2 ± 0.3% compared with no drug control (*p* = 0.03), PCL–CS (*p* < 0.001), and PCL-treated gels (*p* = 0.02) (Figure 7F).

As for the drug solutions from week 3, although the PCL–CS-treated gels contracted less on day 1 and day 2, the contraction area of 5-FU and PCL–CS–5-FU-treated gels became smaller than other groups in the last 5 days (Figure 6C). On day 2, except that the PCL–CS-treated gels contracted less than the no drug control (*p* = 0.01) and PCL-treated gels (*p* = 0.04), there was no statistical significance observed between the other groups (Figure 7C). On day 7, there was also no statistical significance between the implants and no drug control or among the three different implants (Figure 7G).

Lastly, the gels treated with the drug solutions from week 4 exhibited the same trend of matrix contraction as those with the drug solutions from week 2 (Figure 6D). The cells treated with 5-FU showed decreased collagen contraction in comparison to the three different implants and no drug control. There was no statistical significance between the implants and no drug control or among the three different implants on day 2 (Figure 7D). However, the PCL–CS–5-FU-treated gels had lower matrix contraction than that of PCL–CS on day 7 (*p* = 0.03) (Figure 7H).

### Effect of drug loaded implant on the expression of key fibrotic genes

**Figure 8:**
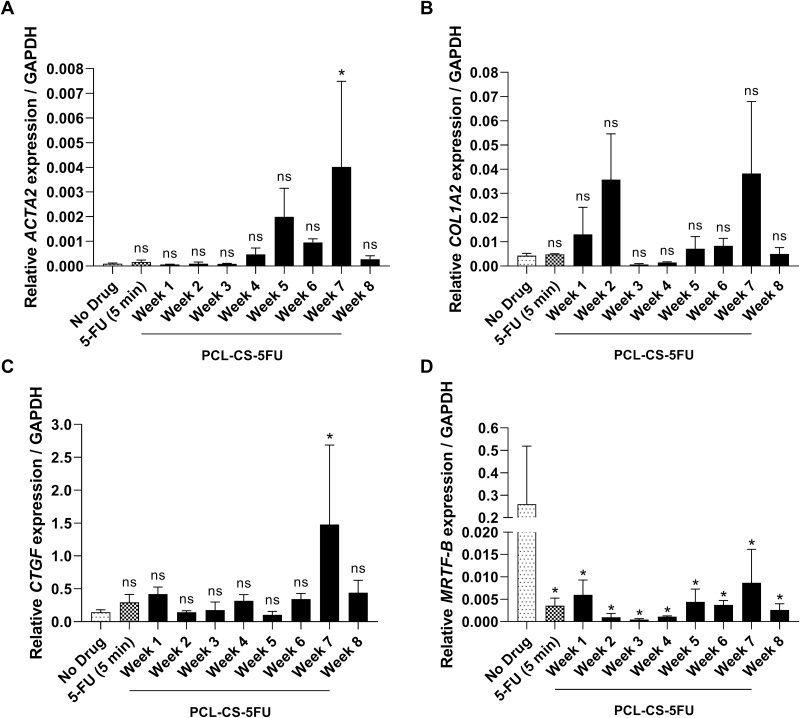
Gene expression of (A) *ACTA2*, (B) *COL1A2*, (C) *CTGF* and (D) *MRTF-B* in human conjunctival fibroblasts after 1-day treatment with no drug control, 5-FU (5 min) and the drug solutions of the PCL–CS–5-FU implant collected from week 1 to week 8. The mRNA expression was normalised against *GAPDH*. Results represent mean ± SEM, *N* = 3. ns, not significant; ^*^p < 0.05.

The expression of key fibrotic genes in human conjunctival fibroblasts after treatment with no drug control, 5-FU and PCL–CS–5-FU solutions from each week over the 8 weeks were measured using RT-qPCR (Figure 8). Compared to no drug control, the cells treated with drug solution from week 7 exhibited an increased expression of *ACTA2* (*p* = 0.03), *COL1A2* (*p* = 0.06) and *CTGF* (*p* = 0.03) genes. Although with no statistical significance compared to no drug control, the cells treated with drug solutions from week 1 and week 3 showed lower *ACTA2* expression (Figure 8A), drug solutions from week 3 and week 4 showed decreased *COL1A2* expression (Figure 8B), and drug solutions from week 2 and week 5 showed lower *CTGF* expression (Figure 8C). Interestingly, all drug solutions from the 8 weeks downregulated the expression of *MRTF-B* gene in comparison to no drug control and all with statistical significance (*p* < 0.05), and the lowest *MRTF-B* gene expression was in week 2, week 3 and week 4 (Figure 8D).

## Discussion

Fibrosis is the main cause of failure of glaucoma filtration surgery and repeated administration of 5-FU is often required postoperatively.^[29]^ However, conventional conventional repeated doses of 5-FU subconjunctival injections pose potential risks of ocular infection, and there is an unmet clinical need to develop an alternative DDS that can sustain the release of 5-FU in conjunctival tissues using a non-invasive method. For this purpose, many micro-devices have been used to achieve the sustained release of 5-FU, such as pHEMA devices,^[30]^ chitosan microtube^[31]^ and poly (lactic acid) disc.^[32]^ However, due to the risks of allograft rejection, most of the allogeneic biological materials are still in the *in vivo* experimental stage.

To achieve a sustained drug release profile in glaucoma treatment, Mohamdeen *et al.* developed 3D-printed drug-eluting contact lenses for timolol.^[33]^ However, the anatomical structure of the eye, namely the presence of structural barriers like the cornea, can present a challenge for the effective delivery of drugs. As an alternative, a minimally invasive implant, which could be directly placed close to the target tissue to release the drug at the site of action, could achieve the same therapeutic efficacy with lower drug concentrations and decreased risk of adverse effects.

In this study, a 3DP technology was used in order to formulate a sustained drug release implant loaded with 5-FU. The physicochemical characterisation of the PCL–CS–5-FU implant has been extensively tested in our previous study,^[25]^ including differential scanning calorimetry, thermal gravimetric analysis, Fourier-transform infrared spectroscopy (FTIR), X-ray micro-computed tomography (µ-CT), X-ray diffraction (XRD) and mechanical behaviour. The PCL–CS–5-FU implant presents excellent thermal stability and good resistance to stretching, which guarantee the safety of the 3DP process at the printing temperature and provide sufficient stability to the host tissue.^[25]^ The advantages of the implant, such as high biocompatibility, slow degradable kinetics and suppression of fibroblast contractility, may therefore be useful as a therapeutic agent to prevent conjunctival fibrosis after glaucoma filtration surgery.

In this study, PCL and chitosan were selected as the drug-eluting polymeric scaffold. The PCL implant was prepared in a *one-step* process and to achieve a good quality scaffold, some bioprinting parameters were changed with the inclusion of chitosan in the implants. The PCL melted at the setting temperature while chitosan was still a powder, therefore the nozzle diameter for PCL–CS implant and PCL–CS–5-FU implant was optimised from 22G to 18G. However, in order to avoid the deposition of too much material due to the increased nozzle diameter and to prevent poor printing quality caused by the increased printing pressure, the printing speed was set to 3.5 mm/s.^[25]^

Under the light microscope, the PCL–CS–5-FU implant had a smooth surface with a certain level of light transmittance. Compared with the original implant form, photographs and light microscopy images demonstrated that the PCL–CS–5-FU implant did not show significant morphological differences after the drug release experiment. PCL is a commonly used base polymer for long-term drug delivery applications as it has a degradation time of 2–3 years, but the degradation rate can be altered by adding CS.^[34]^ In this study, a 3.5% weight decrease was observed, which indicated that the drug is being released and that these scaffolds are biodegradable and will be removed from the body. Scanning electronic microscopy also showed that the PCL, PCL–CS and PCL–CS–5-FU implants are pore-free structures that can prevent the premature drug release. In addition, the smaller-sized implant was designed by the computer-aided design tool to minimise the size of surgical incision and hence reduce trauma to the conjunctiva.

In 2006, Leonard *et al.* reported that PCL can support the attachment of conjunctival epithelial cells *in vitro* and shows good biocompatibility to conjunctival cells.^[35]^ Consistent with these results, we found in our study that PCL, PCL–CS and PCL–CS–5-FU implants did not affect the cell viability and showed good biocompatibility to human conjunctival fibroblasts. This distinct advantage makes the implant a good candidate as a subconjunctival implant material. Besides its biodegradability and biocompatibility properties, the PCL–CS–5-FU implant also exerted a sustained drug release property. In clinic, 5-FU is usually injected subconjunctivally and repeatedly in patients for the first few weeks after trabeculectomy to reduce postoperative subconjunctival fibrosis. In this study, the 5-FU concentration reached and maintained a good level (4–6 μg/ml) during the first 7 weeks, thus we hypothesised that such a drug release profile would enable the PCL–CS–5-FU implant to achieve similar antifibrotic effects compared to conventional repeated 5-FU subconjunctival injections. Furthermore, the PCL–CS–5-FU implant achieved an 8-week sustained drug release, and this prolonged 5-FU release profile suggested that the majority of the drug had been entrapped inside the PCL–CS implant rather than absorbed on the surface. This composite scaffold exhibited the required condition for a drug sustained release system to maintain 5-FU concentration over 2 months to inhibit conjunctival fibrosis effectively.

A fibroblast is a group of highly dynamics cells.^[36]^ Increased fibroblast contraction, associated with the increased extracellular matrix production and scar formation, is usually found in wounded tissues after trauma and surgery.^[37]^ Compared to the no drug control, the PCL–CS–5-FU implant showed the highest decrease in conjunctival fibroblast contractility from week 2, suggesting that the implant would show a significant effect in preventing fibrosis after glaucoma filtration surgery. Another interesting finding is that compared to the PCL only implant, the conjunctival fibroblasts treated with the solution from the PCL–CS implant also showed a significant decrease in fibroblast contraction. A similar result can also be found in highly deacetylated chitosan, which has been reported to inhibit fibroblast-mediated contraction of collagen lattices.^[38]^ A possible explanation is that CS, a multitarget material, can participate in regulating cell cycle and other biological activities to switch fibroblasts from a proliferative, contractile and active state into a quiescent state.^[39, 40]^

To further assess the PCL–CS–5-FU implant ability to suppress conjunctival fibrosis, we measured the expression of key fibrotic genes in human conjunctival fibroblasts after treatment with the PCL–CS–5-FU implant. The myocardin-related transcription factor (MRTF), which is upregulated in many types of fibrotic diseases, is a key fibrotic regulator governing the transcriptional control of extracellular matrix deposition in normal and fibrotic conditions.^[41, 42]^ Our previous studies have shown that liposomal inhibitors generated by nanotechnology sustainably released the MRTF/SRF inhibitor and efficiently suppressed conjunctival fibrosis.^[26]^ In this study, we used the 3D printing technology to manufacture a sustained DDS. Our results show that after 1 week of incubation with the PCL–CS–5-FU implant, the decreased *MRTF-B* expression indicated that the PCL–CS–5-FU implant could effectively inhibit conjunctival fibrosis by downregulating the MRTF/SRF pathway.

## Conclusion

Conjunctival fibrosis is the predominant cause of failure in glaucoma filtration surgery. In this study, a PCL–CS–5-FU implant was manufactured by 3D printing technology, a technology that has started to have applications in ophthalmology^[43]^ to sustainably release 5-FU for long-term use with the aim to prevent postoperative fibrosis and scar formation in conjunctival tissues. By combining PCL and CS, the PCL–CS–5-FU implant not only exhibited desirable biocompatibility and biodegradability, but also released 5-FU over 8 weeks. Furthermore, this sustained 5-FU release system suppressed conjunctival fibrosis which might be achieved by modulating key fibrotic genes. The present study demonstrated that the PCL–CS–5-FU implant has promising potential to be used as a sustained antifibrotic agent in conjunctival tissues and hence to improve the long-term surgical outcomes of glaucoma patients.

## Data Availability

All the data underlying this article are available in the article.
